# Cognitive Impairment as a Putative Mechanism of Self-Management Failure in Chronic Obstructive Pulmonary Disease: A Conceptual Narrative Review

**DOI:** 10.3390/jcm15093550

**Published:** 2026-05-06

**Authors:** Maryam M. Almulhem, Rayan A. Siraj

**Affiliations:** Department of Respiratory Therapy, College of Applied Medical Sciences, King Faisal University, Al-Ahsa 31982, Saudi Arabia; rsiraj@kfu.edu.sa

**Keywords:** cognitive impairment, COPD, self-management, treatment adherence, visuomotor function

## Abstract

**Background/Objectives:** Chronic obstructive pulmonary disease (COPD) management increasingly relies on patient self-management; however, medication non-adherence, inhaler misuse, delayed exacerbation recognition, and suboptimal engagement in pulmonary rehabilitation remain highly prevalent across disease stages. Cognitive impairment is increasingly recognised in this population, particularly in moderate-to-severe disease and in those with greater systemic burden, yet it is most often treated as a descriptive comorbidity rather than a determinant of disease control. **Methods:** This conceptual narrative review synthesises biological, neuropsychological, and clinical evidence to examine the extent to which cognitive impairment contributes to variability in self-management performance and clinical outcomes, and to propose a structured framework linking disease burden, neurocognitive vulnerability, behavioural execution, and downstream outcomes. **Results:** COPD-related processes—including chronic hypoxaemia, hypercapnia, systemic inflammation, oxidative stress, vascular comorbidity, and recurrent exacerbations—provide biologically plausible pathways to neurocognitive vulnerability. Reported deficits in executive function, attention, working memory, processing speed, and visuomotor integration may affect the execution of cognitively demanding tasks central to disease management, including inhaler technique, medication adherence, symptom appraisal, and sustained participation in pulmonary rehabilitation. Across studies, cognitive impairment is consistently associated with inhaler errors, reduced adherence and independence, rehabilitation dropout, impaired symptom recognition, increased healthcare utilisation, functional decline, and mortality. **Conclusions:** Collectively, these findings support the interpretation that cognitive vulnerability may act as an intermediary mechanism through which disease burden translates into behavioural instability and adverse outcomes. Although this framework remains hypothesis-generating, it provides a coherent basis for future longitudinal and interventional studies to formally evaluate the mediating role of cognition in disease management and outcome trajectories.

## 1. Introduction

Chronic obstructive pulmonary disease (COPD) is a leading cause of morbidity and mortality worldwide and represents a substantial and growing public health burden [1,2]. The disease is characterised by persistent airflow limitation, recurrent exacerbations, progressive functional decline, and significant healthcare utilisation. Importantly, COPD rarely occurs in isolation and commonly coexists with comorbid conditions, including cardiovascular disease, metabolic disorders, depression, anxiety, and systemic inflammatory states [3,4]. This multimorbidity further complicates disease management and contributes to poor prognosis, reduced quality of life, and increased hospitalisation risk [5].

Contemporary COPD management increasingly relies on patient-centred self-management strategies [6]. Beyond pharmacological treatment, individuals with COPD are expected to adhere to inhaled therapies, use inhaler devices correctly, monitor symptoms, recognise early exacerbations, implement action plans, titrate oxygen therapy when prescribed, and maintain participation in pulmonary rehabilitation. Effective disease control therefore depends not only on appropriate medical prescribing but also on the patient’s capacity to consistently execute complex health-related behaviours within the context of chronic disease and multimorbidity.

Despite structured self-management programmes and guideline-based interventions, outcomes remain heterogeneous. Medication non-adherence, inhaler misuse, delayed exacerbation recognition, and suboptimal engagement in rehabilitation persist across disease stages [7,8]. These shortcomings are commonly attributed to motivational, educational, psychological, and socioeconomic factors [8]. While these factors undoubtedly contribute, these explanations largely reflect psychosocial and educational models of adherence and implicitly assume intact cognitive systems capable of planning, sequencing, monitoring, and adaptive decision-making. As a result, the potential contribution of neurocognitive vulnerability to behavioural execution remains comparatively underdeveloped within the conceptual framework of COPD self-management.

Cognitive impairment is increasingly recognised among individuals with COPD, particularly in moderate-to-severe disease and in those with greater systemic burden [9,10,11]. Deficits in executive function, working memory, attention, and processing speed have been described [12]. From a biological perspective, COPD-related mechanisms, including chronic hypoxaemia, intermittent hypercapnia, systemic inflammation, vascular comorbidity, and exacerbation-related physiological stress, provide plausible pathways for cerebral vulnerability [13,14]. Executive systems are central to planning, task coordination, error monitoring, and goal-directed behaviour, processes that underpin effective self-management. Although cognitive impairment in COPD has been increasingly described, it has predominantly been examined as a comorbid clinical feature rather than as a functionally relevant determinant of disease management.

Self-management behaviours are inherently cognitively demanding. Proper inhaler use requires sequencing and timing; early detection of symptom deterioration requires sustained attention; implementation of exacerbation action plans requires working memory and anticipatory decision-making [11]. When executive capacity is compromised, the translation of clinical knowledge into consistent behavioural execution may become unreliable. In such contexts, apparent behavioural failure may reflect impaired cognitive execution rather than solely motivational limitations. This distinction is clinically important, as it reframes apparent non-adherence or behavioural inconsistency as a potential manifestation of impaired cognitive execution rather than solely reduced motivation or engagement.

Taken together, these observations support the hypothesis that cognitive impairment may function as an intermediary mechanism linking COPD burden to impaired self-management behaviours and subsequent adverse clinical outcomes. This conceptualisation extends beyond descriptive associations and proposes a structured pathway through which biological disease processes may translate into behavioural instability and clinical deterioration. This conceptual narrative review synthesises biological, neuropsychological, and clinical evidence to examine the extent to which cognitive impairment contributes to variability in self-management performance and clinical outcomes. It proposes a structured framework linking disease burden, neurocognitive vulnerability, behavioural execution, and downstream outcomes.

## 2. Biological Pathways Underlying Cognitive Vulnerability in COPD

COPD is increasingly recognised as a systemic inflammatory and vascular disorder with consequences that extend beyond the respiratory system. Cognitive impairment in COPD is unlikely to be incidental; rather, it may arise from a constellation of interacting biological processes that influence cerebral structure and function. Multiple interrelated mechanisms, including gas exchange abnormalities, oxidative stress, vascular pathology, and recurrent exacerbations, provide a biologically plausible framework linking pulmonary dysfunction to neurocognitive vulnerability. Importantly, these processes offer a mechanistic basis through which disease burden may translate into impaired cognitive capacity, with downstream implications for the execution of self-management behaviours.

### 2.1. Chronic Hypoxaemia

Chronic hypoxaemia represents one of the most biologically plausible mechanisms linking COPD to cognitive impairment [15,16], particularly in moderate-to-severe disease with sustained reductions in arterial oxygen tension. Hypoxia-mediated neuronal injury and reduced activity of oxygen-dependent neurotransmitter systems provide a mechanistic basis for cognitive vulnerability. Broader hypoxia research demonstrates consistent impairment in attention, memory, processing speed, and executive function under both acute and chronic oxygen deprivation. A meta-analysis of nine studies reported a moderate negative correlation between PaO_2_ and cognitive performance (pooled r = 0.405) [17], supporting a measurable association between reduced oxygenation and poorer cognition, although individual studies have reported variable association strengths. Cognitive deficits have been observed in hypoxemic COPD cohorts and appear to increase with disease severity and duration [18], suggesting a cumulative hypoxic burden. While hypoxaemia alone does not fully explain all observed deficits, the high prevalence of oxygen desaturation in COPD provides a biologically plausible pathway through which pulmonary dysfunction may contribute to neurocognitive decline. Such deficits, particularly in attention and executive function, may directly affect the reliability of cognitively demanding self-management behaviours.

### 2.2. Hypercapnia

Hypercapnia represents a related but distinct mechanism that may contribute to cognitive vulnerability in COPD. Chronic CO_2_ retention is common in advanced disease, particularly in patients with severe airflow limitation or respiratory failure [19]. Clinical studies examining the relationship between PaCO_2_ levels and cognitive performance have reported mixed findings. Some studies describe associations with impairments in executive function, attention, verbal memory, and processing speed [20,21], whereas others have not observed a significant correlation [22]. These differences may reflect variation in disease severity, duration of hypercapnia, and physiological adaptation.

From a physiological perspective, sustained hypercapnia can alter cerebral blood flow, disrupt acid–base balance, and influence neuronal function [23,24]. These changes may affect brain regions involved in attention and executive control. Although the precise contribution of hypercapnia to cognitive decline in COPD remains uncertain, its effects on cerebral physiology provide a plausible pathway through which advanced respiratory dysfunction may contribute to domain-specific cognitive impairment, particularly in executive and attentional processes. These domains are central to behavioural regulation, suggesting potential downstream effects on self-management capacity.

### 2.3. Oxidative Stress and Systemic Inflammation

Oxidative stress constitutes a central systemic mechanism linking COPD to cognitive impairment. COPD is characterised by persistent oxidative stress and redox imbalance, with evidence showing reduced antioxidant capacity and increased oxidative injury in patients with COPD compared with healthy controls [25]. Excess reactive oxygen species promote endothelial dysfunction, impair nitric oxide signalling, and disrupt vascular repair, potentially compromising cerebral perfusion. At the cellular level, oxidative stress contributes to mitochondrial dysfunction and DNA damage—processes closely associated with neurodegeneration and cognitive decline. Evidence also suggests that DNA repair mechanisms may be impaired in individuals with cognitive impairment [26], which may amplify vulnerability in COPD populations exposed to chronic oxidative injury. In parallel, oxidative stress sustains chronic inflammatory activation, enhancing microglial-mediated neuronal injury and synaptic dysfunction. Collectively, these vascular, metabolic, and inflammatory processes provide a biologically coherent pathway through which systemic oxidative stress may contribute to progressive cognitive dysfunction in COPD. This systemic mechanism may underlie more diffuse and global patterns of cognitive vulnerability.

### 2.4. Vascular Comorbidity and Cerebral Small Vessel Disease

Vascular comorbidity represents another important pathway linking COPD to cognitive impairment [27]. Cardiovascular disease is highly prevalent in COPD [28], with over half of hospitalised patients demonstrating coexistent vascular conditions, and each disease independently increases the risk of cognitive decline [29]. In the general population, hypertension and diabetes are associated with accelerated decline in executive function and processing speed, and combined vascular risk factors account for a significant proportion of variance in global cognitive performance. Analyses from the Rotterdam Study have demonstrated a higher prevalence of cerebral microbleeds in individuals with COPD, particularly in deep brain regions, and COPD independently predicted their development over time, especially in those with more severe airflow limitation and frequent exacerbations [30]. Cerebral microbleeds, as markers of small vessel disease, were associated with cognitive decline, and neuroimaging findings further support the role of cerebrovascular damage in COPD-related cognitive impairment [31]. Although the cognitive profile in COPD differs from that observed in primary vascular dementia, suggesting that vascular pathology alone is insufficient, vascular comorbidity likely acts as a significant contributor within a broader multifactorial framework, reducing cerebral perfusion and increasing neural vulnerability.

### 2.5. Recurrent Exacerbations

Frequent exacerbations may further amplify cognitive vulnerability in COPD. Acute exacerbations are complex events characterised by infection, hypoxaemia, and substantial physiological and psychological stress. These episodes have been associated with accelerated lung function decline and worse overall clinical outcomes, indicating increased cumulative disease burden. During acute exacerbations, patients with COPD have demonstrated greater cognitive impairment compared with individuals hospitalised for acute heart failure [32], suggesting that exacerbations may precipitate transient neurocognitive deterioration. Although cognitive performance appears to improve within six weeks in many patients [33], recovery is not universal, and the long-term cognitive impact of repeated exacerbations remains uncertain [34]. Given the recurrent nature of exacerbations in advanced disease, repeated acute insults may contribute to cumulative neural stress and progressive cognitive decline over time, potentially accelerating behavioural instability in advanced disease.

### 2.6. Integrated Biological Framework

Taken together, these mechanisms suggest that cognitive impairment in COPD arises from the convergence of multiple interacting biological processes rather than a single pathological driver. Chronic hypoxaemia and hypercapnia may directly impair neuronal metabolism and cerebral autoregulation, while oxidative stress and systemic inflammation promote endothelial dysfunction, mitochondrial injury, and impaired neural repair. Vascular comorbidity introduces additional microvascular compromise, and recurrent exacerbations impose repeated acute physiological stress. These processes likely interact within a multifactorial biological network in which pulmonary dysfunction extends beyond the lung to influence cerebral integrity. Within this framework, neurocognitive vulnerability may represent a biologically grounded consequence of systemic disease burden, with potential downstream effects on behavioural regulation and the execution of self-management tasks. The principal biological pathways discussed above are summarised in Table 1.

## 3. Cognitive Domains Relevant to Self-Management in COPD

Cognition refers to the mental processes through which individuals acquire, interpret, and use information to guide behaviour [35]. In clinical neuropsychology, cognitive functioning is typically described across several domains, including memory, attention, executive function, language, visuospatial ability, and social cognition [12]. These domains support complex daily tasks and functional independence. In chronic diseases such as COPD, effective self-management depends on the coordinated functioning of these cognitive systems, as patients are required to interpret symptoms, follow treatment instructions, and adapt behaviours over time. Impairment in one or more domains may therefore disrupt the consistency and reliability of disease-management behaviours.

Learning and memory involve the ability to encode, store, and retrieve information. These functions allow patients to remember medication schedules, recall inhaler instructions, and follow action plans during symptom worsening. Prospective memory, which supports remembering to perform tasks in the future, is particularly important for daily medication use and symptom monitoring. When memory is impaired, patients may forget medication doses, repeat inhaler errors, or miss early signs of deterioration, reducing the consistency of treatment behaviours. These impairments may undermine prospective adherence and early response to symptom deterioration.

Attention and processing speed support the ability to focus and respond to information efficiently. Sustained attention is needed to complete multi-step tasks such as inhaler use or oxygen management. Alternating attention allows patients to shift between symptom monitoring and daily activities. Processing speed influences how quickly patients interpret changes in breathlessness or fatigue. Slower processing may delay recognition of clinical deterioration and postpone appropriate action.

Executive function includes higher-order cognitive processes such as planning, organisation, decision-making, working memory, and mental flexibility. These functions help individuals regulate goal-directed behaviour and adapt to changing situations. Effective COPD self-management requires patients to integrate medical advice into daily routines and adjust behaviour in response to symptoms. Executive dysfunction may therefore directly impair the ability to plan, sequence, and sustain complex treatment behaviours, making it a critical determinant of self-management stability in COPD [36].

Language abilities include both understanding information and expressing it clearly. Receptive language supports comprehension of medical instructions and educational materials. Expressive language allows patients to describe symptoms and communicate concerns to healthcare providers. Impairment in these areas may lead to misunderstanding treatment instructions or delayed reporting of symptom changes. Although language deficits are less frequently studied in COPD than executive or attentional impairments, they may still influence effective communication, potentially affecting understanding of treatment, adherence, and timely help-seeking [11].

Visuospatial and motor abilities involve visual perception, spatial judgement, and coordination. These skills are important for correct inhaler use, device handling, and oxygen equipment management. Impairment in visuomotor coordination may lead to device errors despite appropriate instruction. As a result, treatment effectiveness may be reduced even when medications are prescribed correctly.

Social cognition and emotional regulation influence insight, motivation, and behavioural consistency. Self-monitoring and emotional control help patients maintain adherence to treatment plans and lifestyle modifications. Difficulties in these areas may reduce engagement in rehabilitation programmes or interfere with decision-making during symptom worsening.

Clinically, cognitive impairment in COPD may present with memory difficulties, repetitive questioning, word-finding problems, impaired decision-making, or reduced functional independence [11]. Visuospatial disturbances and mood changes may also occur. These features are not merely neurological observations; they may directly influence daily disease management. Importantly, cognitive impairment in COPD is often domain-specific rather than global. Subtle deficits, particularly in executive function or attention, may substantially disrupt self-management even in the absence of overt dementia.

A summary of the main cognitive domains and their relevance to COPD self-management is presented in Table 2. The following section examines how impairment in these domains may translate into instability in disease self-management and clinical outcomes.

## 4. Impact of Cognitive Impairment on Self-Management and Clinical Outcomes

Cognitive impairment in COPD is associated with clinically meaningful challenges in disease management [11]. Effective self-management in COPD requires the sustained execution of cognitively demanding behaviours, including correct inhaler use [37], medication adherence [38], symptom monitoring, participation in pulmonary rehabilitation [39], and an appropriate response to exacerbations. These behaviours depend on intact executive function, working memory, attention, processing speed, and visuomotor coordination. When these domains are compromised, even subtly, the translation of medical recommendations into consistent behavioural execution becomes unreliable. Accumulating evidence suggests that cognitive impairment may meaningfully disrupt these self-management processes [40], thereby increasing vulnerability to treatment instability and adverse outcomes.

### 4.1. Inhaler Technique and Procedural Errors

Correct inhaler technique is essential for optimal drug delivery and symptom control. However, inhaler misuse remains highly prevalent, particularly among older individuals with chronic lung disease. Studies report inhaler errors in more than 70% of patients, with critical mistakes occurring in approximately one-third [41]. Cognitive performance has consistently emerged as an independent predictor of inhaler competence [37]. Lower MMSE scores have been associated with worse performance on structured inhaler evaluation scales, even after adjusting for age, education, and sex. A graded relationship has been observed, such that incremental improvements in cognitive score correspond to reductions in inhaler error burden [42].

In inhaler-naïve patients with COPD, reduced neuropsychological test performance correlates with increased training time, higher error frequency, and poorer device mastery at follow-up [43]. Executive dysfunction and dyspraxia, in particular, impair the ability to sequence inhaler steps correctly [36]. Systematic review evidence confirms that cognitively impaired individuals are more likely to demonstrate inhaler incompetence and require assistance in daily management [37]. While supervised training may temporarily improve performance, this is likely to reflect compensatory support rather than preserved autonomous ability. Collectively, these findings suggest that inhaler misuse may partly reflect underlying cognitive vulnerability, particularly in domains related to sequencing, motor planning, and executive control, rather than solely deficits in knowledge or training.

### 4.2. Medication Adherence and Behavioural Regulation

Medication adherence in COPD requires prospective memory, organisational capacity, and behavioural consistency. Cognitive impairment has been associated with reduced independence in daily activities and increased need for assistance in treatment management. Evidence indicates that cognitively impaired patients are less likely to adhere consistently to prescribed regimens [37,39]. Forgetting doses, misunderstanding instructions, and difficulty integrating medications into daily routines are frequent contributors to non-adherence [44].

The clinical implications of poor adherence are substantial. Suboptimal adherence is associated with increased exacerbation risk, higher hospitalisation rates, unnecessary escalation of pharmacotherapy, increased healthcare expenditure, and reduced quality of life [44]. Notably, only 40–60% of patients with COPD demonstrate adequate adherence based on pharmacy refill and self-report data, and correct execution of the metered-dose inhaler technique is achieved by a minority [45]. When cognitive impairment is present, these vulnerabilities may be amplified. Memory deficits may lead to missed or duplicated doses, while executive dysfunction may impair planning and sustained behavioural engagement. Thus, cognitive impairment represents a mechanistically relevant barrier to consistent pharmacological management, primarily through its effects on memory, planning, and behavioural regulation.

### 4.3. Pulmonary Rehabilitation Participation and Completion

Pulmonary rehabilitation (PR) is a cornerstone intervention that improves exercise capacity, symptom burden, and health-related quality of life. Successful participation requires planning, sustained attention, impulse control, and adherence to structured behavioural routines over time. Evidence indicates that cognitive impairment increases the likelihood of PR dropout. In a cohort of 157 patients, individuals with cognitive impairment demonstrated significantly higher dropout rates compared to cognitively intact participants (23% versus 10%) [39].

Importantly, among those who completed the programme, improvements in functional outcomes were comparable regardless of cognitive status. This suggests that cognitive impairment does not necessarily diminish physiological responsiveness to rehabilitation but may impair behavioural persistence and engagement. Executive dysfunction may interfere with attendance planning, motivation, and adherence to prescribed exercise routines. Therefore, cognitive impairment may function as a barrier to sustained participation rather than a measure of therapeutic efficacy, highlighting that behavioural engagement, rather than physiological capacity, may be the primary limiting factor in cognitively vulnerable patients.

### 4.4. Symptom Recognition and Exacerbation Response

Effective COPD management requires timely recognition of symptom deterioration and appropriate activation of action plans. This process relies on attention, processing speed, memory recall, and decision-making capacity. Mild cognitive impairment has been associated with reduced symptom recall, suggesting that patients may fail to detect early warning signs of exacerbation [46]. Slowed cognitive processing and impaired executive function may delay help-seeking behaviour or correct implementation of rescue therapy [47].

Such delays are clinically significant. Exacerbations are associated with accelerated lung function decline, hospitalisation, and mortality. If cognitive impairment interferes with timely symptom appraisal and response, it may contribute to delayed intervention, increased exacerbation severity, and greater healthcare utilisation. Although longitudinal mediation studies remain limited, the behavioural plausibility and consistency of observational findings strongly support this pathway.

### 4.5. Diagnostic Accuracy and Healthcare Communication

Cognitive impairment may also influence the accuracy of clinical assessment and the quality of therapeutic communication [48]. Spirometry requires comprehension of instructions, sustained attention, and coordinated effort across repeated manoeuvres [49]. Cognitive deficits may result in suboptimal performance, potentially leading to inaccurate estimations of lung function and misclassification of disease severity. Similarly, maximal exercise testing requires an understanding of effort expectations and task persistence [50]; impaired cognition may lead to premature termination and underestimation of functional capacity.

Communication challenges further compound these risks [51]. Cognitive dysfunction may reduce the ability to understand complex medical explanations, ask clarifying questions, or engage in shared decision-making. Misunderstandings between patient and clinician may contribute to treatment refusal or inconsistent adherence. These factors suggest that cognitive impairment may influence not only self-management behaviours but also the reliability of disease assessment and therapeutic alignment.

### 4.6. Integrative Synthesis

Across multiple domains, cognitive impairment in COPD is consistently associated with inhaler misuse, reduced adherence, increased need for assistance, higher dropout from pulmonary rehabilitation, impaired symptom recognition, and potential inaccuracies in disease assessment. These effects extend beyond isolated neuropsychological deficits and translate into tangible disruptions in the execution of self-management behaviours.

Although much of the evidence is derived from observational and cross-sectional studies, the consistency of findings across behavioural and functional domains supports a meaningful relationship between cognitive vulnerability and treatment instability [52]. Importantly, these associations align with domain-specific cognitive deficits, particularly in executive function, attention, and memory, which are central to behavioural regulation and task execution.

However, important limitations remain. Existing studies are often based on small samples, use heterogeneous cognitive assessment tools, and vary in their adjustment for confounding factors such as age, education, depression, and comorbidity [37]. Moreover, the predominance of cross-sectional designs limits causal inference, and the temporal relationship between cognitive decline, disease progression, and behavioural disruption remains incompletely understood.

Despite these limitations, the convergence of biological plausibility, domain-specific cognitive deficits, and consistent behavioural associations provides a coherent basis for considering cognitive impairment as a mechanistically relevant contributor to self-management instability in COPD. Future longitudinal studies incorporating formal mediation analyses are required to determine whether cognitive impairment functions as an intermediary pathway linking disease severity to clinical outcomes.

## 5. Cognitive Impairment as an Intermediary Link Between COPD Severity and Clinical Outcomes

Building on the preceding synthesis, this Section integrates existing evidence into a conceptual model in which cognitive impairment may represent an intermediary mechanism linking COPD burden to behavioural instability and clinical outcomes. Within this framework, disease severity may influence outcomes both directly and indirectly through its effects on neurocognitive function and behavioural execution. Although this interpretation remains hypothetical due to limited formal mediation analyses, it provides a structured basis for understanding the interaction between biological vulnerability, cognitive dysfunction, and self-management performance [37].

### 5.1. COPD Severity and Cognitive Vulnerability

Evidence indicates that cognitive impairment in COPD is associated with markers of disease burden, supporting its role as a potential intermediary between pulmonary dysfunction and adverse outcomes. Cross-sectional studies report a higher prevalence of mild cognitive impairment among patients with moderate-to-severe COPD compared with healthy controls [53], with a predominance of non-amnestic deficits affecting attention and executive function—domains critical for behavioural regulation.

Studies examining graded hypoxaemia demonstrate a dose–response relationship between oxygenation status and cognitive performance, with higher rates of impairment observed in patients with more severe gas exchange abnormalities [22]. Although the proportion of shared variance is modest, multivariate analyses confirm that oxygenation remains independently associated with neuropsychological deficits after adjusting for age and education, suggesting that hypoxaemia contributes meaningfully, though not exclusively, to cognitive vulnerability. The heterogeneity of affected domains further implies that multiple interacting mechanisms—including systemic inflammation, vascular dysfunction, and cumulative disease burden—may converge on neural systems governing attention and executive control.

Longitudinal evidence strengthens this association. Severe COPD, defined by oxygen use or significant airflow limitation, has been linked to accelerated cognitive decline over time [54]. Similarly, patients with greater functional limitation have demonstrated increased risk of cognitive impairment compared with those with milder disease [27]. Population-based studies further indicate that COPD is associated with an increased risk of incident cognitive impairment, with evidence of a duration-dependent effect [55]. Collectively, these findings suggest that cognitive vulnerability evolves progressively in relation to cumulative disease exposure rather than representing a static comorbidity. While causality cannot be established, these associations satisfy a key condition for conceptualising cognition as a plausible intermediary between disease burden and downstream clinical outcomes.

Taken together, the convergence of prevalence data, hypoxaemia-gradient findings, and longitudinal risk estimates supports the premise that increasing COPD severity is associated with heightened cognitive vulnerability. While these associations do not establish causation, they satisfy an essential condition for conceptualising cognition as a potential intermediate link between disease burden and downstream clinical outcomes.

### 5.2. Cognitive Impairment and Downstream Clinical Consequences in COPD

Accumulating evidence suggests that cognitive impairment in COPD is clinically consequential. Across diverse populations and study designs, cognitive vulnerability has been associated with increased mortality risk, greater hospitalisation burden, impaired recovery following exacerbations, and broader functional decline.

Recent data indicate that individuals with coexisting COPD and low cognitive performance have substantially higher respiratory-related mortality risk compared with those with COPD alone [56]. Importantly, low cognitive performance in isolation does not independently predict respiratory mortality, suggesting that cognition may amplify disease-specific vulnerability. Earlier prospective studies have similarly identified domain-specific deficits—particularly in visuospatial and executive function—as independent predictors of mortality [57], whereas global screening tools such as the MMSE have shown inconsistent prognostic value.

Beyond mortality, cognitive impairment is associated with increased healthcare utilisation. Individuals with both COPD and cognitive impairment experience higher rates of respiratory-related and all-cause hospitalisations, as well as increased risk of death. These findings suggest that cognitive vulnerability may compound the underlying respiratory risk profile, partly by impairing behavioural regulation and responses to disease progression.

Acute exacerbations represent a particularly vulnerable period [58]. Patients hospitalised for COPD exacerbations consistently demonstrate worse cognitive performance than stable outpatients [34], with impairments observed across executive, attentional, and visuospatial domains. Cognitive dysfunction during exacerbation is associated with poorer health status and longer hospital length of stay. Importantly, follow-up studies indicate that cognitive performance does not uniformly normalise after clinical recovery, raising concern that exacerbations may either unmask latent deficits or accelerate underlying neurocognitive decline. Recurrent episodes of hypoxaemia, systemic inflammation, and metabolic stress may therefore contribute to cumulative neurological burden over time.

Population-based studies further support the link between respiratory dysfunction and cognitive risk. Reduced pulmonary function indices, including FEV_1_, FVC, and peak expiratory flow, are associated with increased risk of cognitive impairment and dementia [59]. Individuals with lung disease exhibit higher combined risks of cognitive impairment compared with those without respiratory disease [59]. Community-based studies similarly report a higher prevalence of cognitive impairment among individuals with advanced airflow limitation [60]. These findings suggest that chronic respiratory dysfunction may influence long-term neurocognitive trajectories through mechanisms such as hypoxaemia, vascular injury, oxidative stress, and systemic inflammation.

Cognitive impairment also extends to functional domains. Executive and memory deficits have been associated with impaired balance, reduced dexterity, and diminished dual-task performance. Although global exercise capacity may not consistently differ, tasks requiring simultaneous cognitive and motor engagement reveal reduced reserve, with implications for physical independence and safety.

Collectively, these findings indicate that cognitive impairment in COPD is associated with adverse outcomes across multiple domains, including survival, hospitalisation, exacerbation recovery, and functional performance. These associations are most evident in patients with advanced disease and domain-specific deficits, particularly in executive and visuospatial function, reinforcing the view that cognitive impairment may represent a clinically meaningful determinant of disease trajectory.

### 5.3. Cognitive Impairment as a Putative Mediating Mechanism

The cumulative evidence supports consideration of cognitive impairment as a putative intermediary mechanism linking disease severity to behavioural instability and adverse clinical outcomes. This interpretation is supported by three converging observations: the increased prevalence of cognitive vulnerability in COPD, the disruption of self-management behaviours associated with domain-specific cognitive deficits, and the consistent association between cognitive impairment and adverse outcomes.

COPD is characterised not only by airflow limitation but also by systemic manifestations, including hypoxaemia, inflammatory activation, vascular comorbidity, and recurrent exacerbatory stress. These processes may exert cumulative effects on neural integrity, particularly within frontal–subcortical networks governing executive control and behavioural regulation. Impairment in these domains has direct relevance for the execution of complex self-management behaviours. Even modest deficits in executive or attentional processes may destabilise disease control by disrupting the consistency of behavioural execution.

Within this framework, behavioural inconsistency should not be interpreted solely as a function of motivational, educational, or psychosocial factors, but also as a potential manifestation of impaired cognitive capacity. This perspective provides explanatory coherence for the observed heterogeneity in COPD trajectories among individuals with comparable physiological impairment, where variability in cognitive reserve may influence the stability of behavioural implementation.

This conceptualisation does not position cognitive impairment as an isolated determinant of outcome. Rather, COPD trajectories reflect the interaction of physiological severity, multimorbidity, psychological factors, social context, and healthcare systems. Within this multidimensional framework, cognition may function as a rate-limiting determinant influencing the extent to which therapeutic interventions are effectively implemented.

**Figure 1:**
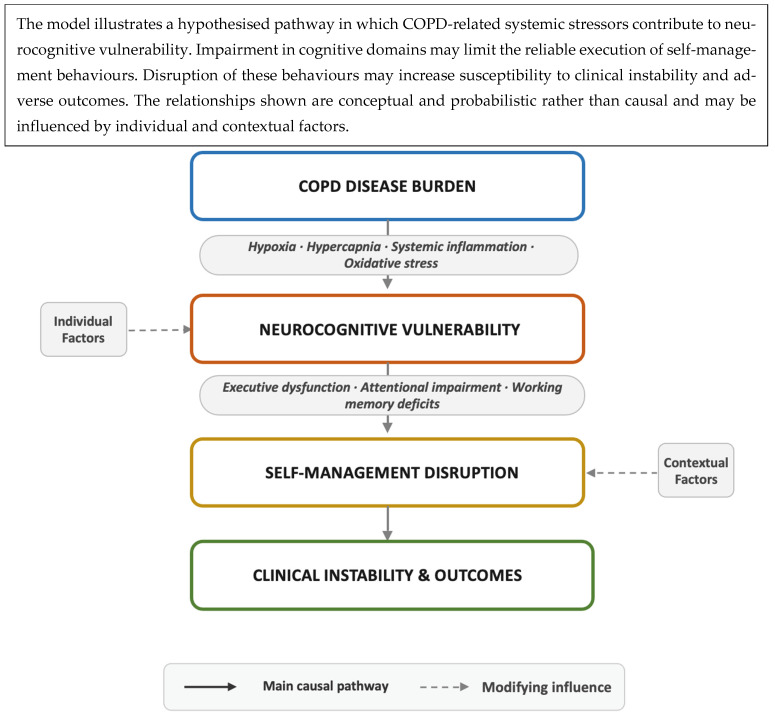
Proposed conceptual pathway linking COPD burden, neurocognitive vulnerability, self-management disruption, and clinical outcomes. COPD burden (e.g., hypoxia, hypercapnia, systemic inflammation, oxidative stress) contributes to neurocognitive vulnerability, characterised by domain-specific impairments such as executive dysfunction, attentional deficits, and working memory impairment. These impairments disrupt self-management behaviours, leading to clinical instability and adverse outcomes. Solid arrows indicate the main pathway, while dashed arrows represent modifying influences. Relationships are probabilistic and hypothesis-generating.

Although definitive mediation testing remains limited, the convergence of biological plausibility, domain-specific cognitive deficits, and consistent behavioural and outcome associations supports the positioning of cognitive impairment as a mechanistically meaningful intermediary in COPD management. Accordingly, a conceptual model is proposed in which COPD severity may contribute to neurocognitive vulnerability; neurocognitive vulnerability may constrain the execution of self-management behaviours; and impaired behavioural execution increases the likelihood of clinical instability and adverse outcomes (Figure 1). This framework is probabilistic rather than deterministic, acknowledges the influence of psychological, socioeconomic, and healthcare system factors, and provides a structured foundation for future longitudinal and interventional research.

## 6. Discussion

### 6.1. Conceptual Contribution

This review synthesises biological, neuropsychological, and clinical evidence to propose a structured conceptual framework linking COPD burden, neurocognitive vulnerability, disruption of self-management behaviours, and downstream clinical outcomes. Although cognitive impairment in COPD has been recognised for several decades and associated with key aspects of disease management, including inhaler technique, medication adherence, and rehabilitation participation, these relationships have largely been examined in isolation. The present review integrates these observations into a unified pathway in which cognitive vulnerability contributes to variability in the execution of self-management behaviours.

Importantly, this framework is hypothesis-generating rather than causal. It is derived from converging empirical evidence rather than direct mediation analyses. By integrating biological plausibility, domain-specific cognitive deficits, and behavioural and outcome associations, this review provides a structured perspective for understanding how systemic disease processes may translate into clinical instability and offers a foundation for future longitudinal and interventional research.

### 6.2. Clinical and Practical Implications

The clinical implications of this framework are substantial. Contemporary COPD management emphasises patient education, behavioural reinforcement, and adherence counselling, implicitly assuming intact cognitive systems capable of translating instruction into sustained action. If cognitive vulnerability constrains behavioural execution, educational interventions alone may be insufficient for a clinically meaningful subset of patients.

Incorporating brief, domain-sensitive cognitive screening—particularly targeting executive function, attention, and working memory—into routine respiratory assessment may help identify individuals at risk of self-management instability. Tools such as Trail Making Test Parts A and B offer complementary sensitivity to processing speed and executive control and are feasible in outpatient settings [61]. Common screening tools such as the Montreal Cognitive Assessment (MoCA) and the Mini-Mental State Examination (MMSE) may also be used, with the MoCA generally demonstrating greater sensitivity to executive dysfunction [11]. However, no COPD-specific thresholds have been established, and results should be interpreted cautiously in the context of age, education, and comorbidity. However, performance should be interpreted cautiously, as it may be influenced by age, education, and motor impairment, which are common in COPD populations [62].

Interventions tailored to cognitive capacity, including repetition-based inhaler training, simplified medication regimens, caregiver involvement, and external cueing strategies, may help mitigate the functional impact of executive dysfunction. More broadly, recognising cognition as a rate-limiting determinant supports a shift from compliance-based models toward capacity-informed approaches to COPD care.

### 6.3. Research Directions and Testable Hypotheses

The proposed framework generates several testable hypotheses. Longitudinal studies are needed to evaluate whether cognitive impairment mediates the relationship between COPD severity and adverse outcomes through disruption of self-management behaviours. Approaches such as structural equation modelling, time-lagged analyses, and formal mediation testing could clarify whether neurocognitive vulnerability temporally precedes behavioural instability and whether behavioural disruption explains the association between cognition and outcomes such as hospitalisation.

Interventional studies examining whether cognitive-support strategies improve adherence, pulmonary rehabilitation completion, or exacerbation response would provide mechanistic validation. Neuroimaging and biomarker research may further identify neural and inflammatory correlates of behavioural instability. Importantly, future studies should distinguish between global cognitive impairment and domain-specific deficits, particularly in executive function, which may be more clinically and prognostically relevant.

Despite these directions, several important gaps remain, including the limited availability of longitudinal and mediation studies, heterogeneity in cognitive assessment methods, and the scarcity of interventional research targeting cognitive impairment in COPD populations. Addressing these gaps will be essential to validate the proposed framework and support its translation into clinical practice.

### 6.4. Broader Conceptual Implications

Beyond immediate clinical implications, this framework contributes to a broader reconceptualisation of heterogeneity in COPD trajectories. Patients with similar spirometric impairment often exhibit markedly different patterns of exacerbation frequency, healthcare utilisation, and treatment response, suggesting that factors beyond lung function influence disease progression.

Cognitive reserve and executive capacity may partially explain this variability by influencing the consistency with which therapeutic behaviours are implemented. This perspective does not diminish the role of psychological, social, or healthcare system factors but situates cognition as an interacting determinant within a multidimensional disease model. Integrating neurocognitive vulnerability into existing frameworks of COPD multimorbidity may therefore enhance risk stratification and support more individualised care strategies.

Impaired self-management in COPD should not be attributed to cognitive impairment alone. A range of complementary factors, including depression [63], anxiety [64], frailty [65], low health literacy [66], socioeconomic disadvantage [67], and multimorbidity [68], may independently or interactively contribute to behavioural instability. These factors may influence motivation, comprehension, physical capacity, and access to care, thereby affecting the execution of self-management behaviours. Accordingly, the proposed framework should be interpreted as complementary rather than exclusive, positioning cognitive impairment as one of several interacting mechanisms within a broader biopsychosocial model of COPD self-management.

As a narrative review, this work has inherent methodological limitations. It does not follow a predefined systematic search strategy or formal study selection process, which may introduce selection bias and limit reproducibility. However, this approach allows for conceptual integration across biological, neuropsychological, and clinical domains. Accordingly, the proposed framework should be interpreted as a structured, hypothesis-generating synthesis rather than a definitive model.

## 7. Conclusions

In summary, converging biological and behavioural evidence supports a coherent pathway in which COPD severity may contribute to neurocognitive vulnerability, and neurocognitive vulnerability, in turn, may shape the stability of self-management behaviours. Cognitive impairment appears to occupy a structurally meaningful position within this pathway, functioning as a potential mediator of clinical instability rather than merely a descriptive comorbidity. Recognising cognitive capacity as a determinant of self-management provides a conceptual basis for integrating domain-sensitive screening into respiratory care. Although longitudinal mediation studies are required to establish causality, the existing evidence offers a strong foundation for further evaluation of this model in both research and clinical practice.

## Figures and Tables

**Table 1:** Biological pathways linking COPD to cognitive impairment.

Biological Pathway	COPD-Related Trigger	Cerebral Mechanism	Cognitive Domains Affected
Chronic Hypoxemia	Sustained reductions in arterial oxygen tension in moderate-to-severe COPD	Hypoxia-mediated neuronal injury; reduced activity of oxygen-dependent neurotransmitter systems	Attention, processing speed, and executive function
Hypercapnia	Chronic CO_2_ retention in advanced COPD	Disruption of cerebral autoregulation; respiratory acidosis; inflammatory activation and neurochemical alteration	Executive function, complex attention, verbal memory, and processing speed
Oxidative Stress and Systemic Inflammation	Persistent redox imbalance and systemic inflammatory activation	Endothelial dysfunction; mitochondrial dysfunction; DNA damage; microglial-mediated neuronal injury	Global cognitive vulnerability
Vascular Comorbidity and Cerebral Small Vessel Disease	High prevalence of cardiovascular disease and vascular risk factors	Microvascular compromise; cerebral microbleeds; reduced cerebral perfusion	Processing speed, executive function, global cognitive performance
Recurrent Exacerbations	Acute infection, hypoxemia, and physiological stress episodes	Transient neurocognitive deterioration; cumulative neural stress from repeated insults	Transient impairment; possible cumulative decline with repeated events

This table summarises the principal biological pathways through which COPD may contribute to neurocognitive vulnerability. For each pathway, the COPD-related trigger, proposed cerebral mechanism, and cognitive domains most commonly reported in the literature are outlined. These mechanisms are interrelated and reflect a multifactorial framework rather than isolated causal effects.

**Table 2:** Cognitive Domains and Their Relevance to Self-Management in COPD.

Cognitive Domain	Core Functions	Relevance to COPD Self-Management	Potential Consequences of Impairment
Learning and Memory	Encoding, short- and long-term memory, prospective memory, recall	Remembering medication schedules, inhaler steps, oxygen settings, and action plans	Missed doses, repeated inhaler errors, failure to recognise early symptom worsening
Attention and Processing Speed	Sustained attention, alternating attention, information processing speed	Following multi-step instructions, monitoring symptoms, responding to clinical changes	Delayed response to exacerbations, incomplete task execution, poor adherence consistency
Executive Function	Planning, organising, decision-making, working memory, mental flexibility, inhibition	Implementing action plans, adapting behaviour, integrating medical advice into daily routines	Poor treatment adherence, difficulty adjusting therapy, inability to manage complex regimens
Language	Comprehension, word finding, fluency, expressive and receptive language	Understanding medical instructions and communicating symptoms to providers	Misinterpretation of treatment plans, delayed help-seeking
Visuospatial and Motor Function	Visual perception, visuoconstruction, perceptual–motor coordination	Correct inhaler handling, device assembly, oxygen equipment management	Device misuse, reduced treatment effectiveness
Social Cognition and Emotional Regulation	Self-awareness, self-monitoring, emotion recognition, self-control	Sustained engagement in rehabilitation, lifestyle modification, and adherence motivation	Reduced participation in rehabilitation, inconsistent behavioural regulation

This table summarises the principal neuropsychological domains, their core functional components, and their relevance to key self-management tasks in COPD. It illustrates how domain-specific cognitive impairments may interfere with medication adherence, inhaler technique, symptom monitoring, rehabilitation engagement, and overall disease control.

## Data Availability

No new data were created or analysed in this study.
